# Analysis of 24-h Rhythm in Ventricular Repolarization Identifies QT Diurnality As a Novel Clinical Parameter Associated with Previous Ventricular Arrhythmias in Heart Failure Patients

**DOI:** 10.3389/fphys.2017.00590

**Published:** 2017-08-15

**Authors:** Bastiaan C. Du Pre, Linda W. Van Laake, Matthias Meine, Jeroen F. Van der Heijden, Pieter A. Doevendans, Marc A. Vos, Toon A. B. Van Veen

**Affiliations:** ^1^Division of Heart and Lungs, Department of Medical Physiology, University Medical Center Utrecht Utrecht, Netherlands; ^2^Division of Heart and Lungs, Department of Cardiology, University Medical Center Utrecht Utrecht, Netherlands

**Keywords:** circadian, repolarization, QT, ventricular arrhythmia, sudden cardiac death, rhythm

## Abstract

**Introduction:** Cardiac repolarization abnormalities are among the major causes of ventricular arrhythmias and sudden cardiac death. In humans, cardiac repolarization duration has a 24-h rhythm. Animal studies show that this rhythm is regulated by 24-h rhythms in ion channel function and that disruption of this rhythm leads to ventricular arrhythmias. We hypothesized that 24-h rhythms in QT duration can be used as a predictor for sudden cardiac death and are associated with ventricular arrhythmias. Secondly, we assessed a possible mechanistic explanation by studying the putative role of hERG channel dysfunction.

**Materials and Methods:** In 2 retrospective studies, measures of the 24-h variation in the QT and QTc intervals (QT and QTc diurnality, QTd and QTcd, respectively) have been derived from Holter analyses and compared between groups: 1) 39 post-infarct patients with systolic heart failure (CHF: *EF* < 35%), of which 14 with, and 25 without a history of ventricular arrhythmias and 2) five patients with proven (LQTS2) and 16 with potential (Sotalol-induced) hERG channel dysfunction vs. 22 controls.

**Results:** QTd was two-fold higher in CHF patients with a history of ventricular arrhythmias (38 ± 15 ms) compared to CHF patients without VT (16 ± 9 ms, *p* = 0.001). QTd was significantly increased in LQT2 patients (43 ± 24 ms) or those treated with Sotalol (30 ± 10 ms) compared to controls (21 ± 8 ms, *p* < 0.05 for both).

**Discussion:** QT diurnality presents a novel clinical parameter of repolarization that can be derived from Holter registrations and may be useful for identification of patients at risk for ventricular arrhythmias.

## Introduction

Sudden cardiac death (SCD), often caused by ventricular arrhythmias, is a major health problem, which affects 1 in every 500 persons per year (Niemeijer et al., 2015). Several parameters to identify patients at risk for SCD have been proposed in the last decades. In patients with primary prophylactic implantable cardioverter/defibrillator (ICD) therapy however, the number needed to treat is still high [11 in MADIT2 (Moss et al., 2002) and 14 in SCD-HeFT (Bardy et al., 2005)] and possibly underestimated. Criteria that can decrease the number of patients needed to treat will reduce the ICD burden, which consists of regular ICD device checks, battery changes, occupational/lifestyle restrictions and the risk of surgical complications or unnecessary shocks (Szwejkowski et al., 2015). Furthermore, better risk prediction will make ICD therapy more cost effective. Clearly, there is a high demand for better SCD prediction in patients currently treated with a primary prophylactic ICD.

24-h rhythms are biorhythms present within many cardiac electrophysiological parameters, including QRS duration, QT interval, and heart rate variability (HRV; Nakagawa et al., 1998). These rhythms are regulated by diurnal changes in ion channel function, such as the hERG channel (Schroder et al., 2015). In addition, 24-h rhythmicity has been linked to the incidence and pathophysiology of ventricular arrhythmias and SCD, which display an increased incidence in the morning. In animal studies, disruption of the normal 24-h rhythm leads to a severely depressed functioning of hERG channels conducting the repolarizing current I_Kr_ and a concomitant increased susceptibility to ventricular arrhythmias (Jeyaraj et al., 2012; Schroder et al., 2015). Ventricular repolarization is humans however, significantly differs between humans and animals (ICH S7B guidelines, 2015). So far, it remains unknown whether the presence and nature of 24-h rhythms in electrophysiological parameters in humans possess predictive value for the manifestation of ventricular arrhythmias and SCD.

We hypothesized that disruption of physiological 24-h rhythms in ventricular repolarization duration is associated with ventricular arrhythmias. In the current retrospective study, we therefore compared 24-h rhythms in QT interval duration between heart failure patients with and without history of ventricular arrhythmias. Our data suggest that the amplitude of the 24-h rhythms in QT and QTc interval (QT and QTc diurnality, QTd, and QTcd, respectively) is not only associated with ventricular arrhythmias, but also linked to disturbed functioning of I_Kr_.

## Materials and methods

The study consists of two analyses. In the first analysis, we compared 24-h rhythms in QT interval duration between heart failure patients with and without history of ventricular arrhythmias. In this first part, we introduce two novel parameters QT and QTc diurnality, which are measures for the amount of 24-h variation (amplitude) in the QT and QTc interval (**Figure 2**). In the second analysis, we studied whether these parameters are related to hERG channel functionality. We compared QTd and QTcd levels between subjects with genetic or drug-induced hERG channel disfunction and controls.

### Patient selection

#### 24-h rhythms in repolarization and ventricular arrhythmias (*n* = 39)

Patients with systolic heart failure (HFrEF; heart failure NYHA class II and above with an LVEF <35%) and a history of a myocardial infarction were included from three different sources: (1) Patients of the UMC Utrecht participating in the EUTrigTreat study (Seegers et al., 2012). Holter analysis was part of the EUTrigTreat study protocol and already performed in all patients before the start of the current study. (2) Patients of the UMC Utrecht who received a guideline-indicated ICD implantation as primary prevention (*EF* < 35%) between 2009 and 2011 (Zipes et al., 2006), and that received a Holter registration after ICD implantation. (3) Patients who attended the outpatient clinic of the UMC Utrecht between September and November 2014 and had a history of ventricular arrhythmias. In patients from the third source (outpatient clinic UMC Utrecht, all with ICD upon inclusion) Holter analysis was performed after informed consent was obtained.

Exclusion criteria for all patients in this study were: (1) Use of antiarrhythmic drugs (all classes, slow–release beta-blockers excluded) during or in the 6 weeks before the Holter registration. (2) Supraventricular arrhythmias during Holter registration. (3) Myocardial infarction in the 3 years prior to Holter registration.

All patients included in this study were subdivided in two groups, based on history of ventricular arrhythmias. VT+ means that the arrhythmia occurred after the moment that EF was reduced below 35%. All VT+ were confirmed by either a electrocardiogram or manual check of the ICD-recording.

#### 24-H rhythms in repolarization and hERG channel functionality

##### Genetic hERG channel dysfunction: LQTS2 (*n* = 5)

Patients clinically diagnosed with LQTS type II carrying a loss-of function mutation of the KCNH2 gene encoding the protein that constitutes the hERG channel, which attended the outpatient clinic of the UMC Utrecht and received a 24-h electrocardiogram recording (Holter) between 2009 and 2015, were included.

##### Drug-induced hERG channel dysfunction: sotalol users (*n* = 16) (and controls, *n* = 22)

Patients who successfully underwent pulmonary vein isolation for treatment of atrial fibrillation, and received a Holter between May and September 2015, were screened. Subjects that used Sotalol for at least 2 weeks or were devoid of anti-arrhythmic medication for at least 2 weeks (controls) during their Holter were included. Exclusion criteria were the use of other anti-arrhythmic drugs (all classes, beta blockers excluded), a left ventricular ejection fraction (LVEF) of <45%, and presence of supraventricular arrhythmia during Holter registration.

A summary of patient source and stratification is provided in Table 1.

**Table 1:** Patient source and stratification.

**Source**	**Analysis 1**		**Analysis 2**		
	**VT/VF−**	**VT/VF+**	**Control**	**Sotalol**	**LQTS2**
EUTrigTreat	11	7			
ICD Implantation Database 2009-2011	14	2			
Outpatient Clinic Cardiology: Sep–Nov 2014		5			
Outpatient Clinic Cardiology: All					5
Holter Database: PVI Patients May–Sep 2015			22	16	

*ICD, Implantable cardioverter/defibrillator; PVI, Pulmonary Vein Isolation; VT/VF, Ventricular Tachycardia/Ventricular Fibrillation*.

The investigation conforms with the principles outlined in the Declaration of Helsinki. The Medical Ethical Committee of the UMC Utrecht approved both retrospective studies (reference #WAG/mb/15/036026).

### Patient characteristics

Age, gender, medication use, echocardiography parameters, NYHA classification, comorbidities (diabetes mellitus, renal failure), medication history, and history of ventricular arrhythmias at the time of the Holter were collected from the electronic patient file. Ventricular arrhythmias (Ventricular fibrillation or sustained ventricular tachycardia) were only qualified as such when recorded on an electrocardiogram (ECG) lasting at least 30 s. Ventricular arrhythmias detected by the ICD were manually checked for accuracy.

**Figure 1:**
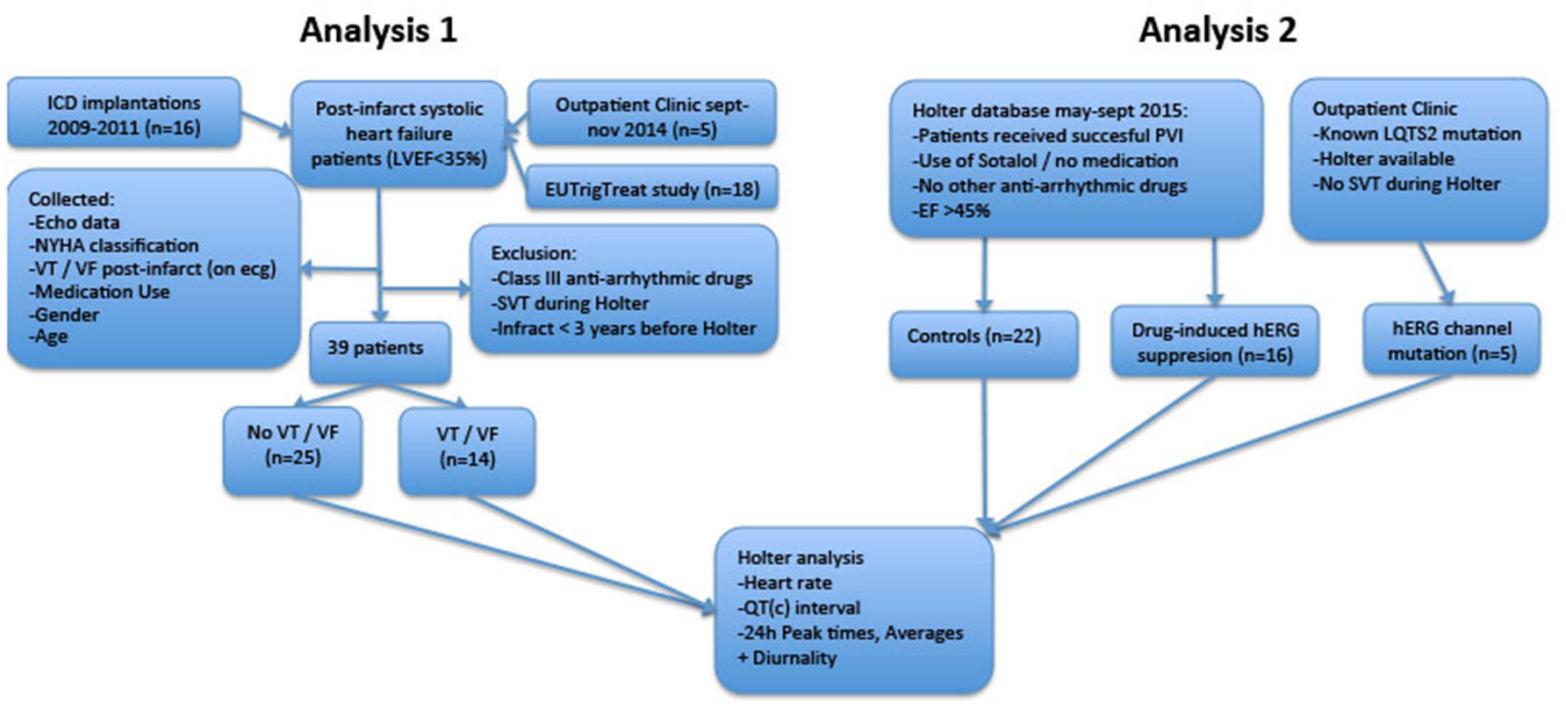
Flowchart of retrospective analyses. LVEF, Left Ventricular Ejection Fraction; ICD, Implantable Cardioverter/Defibrillator; LQTS2, Long QT Syndrome Type 2; PVI, Pulmonary Vein Isolation; SVT, Supraventricular Arrhythmia; VT/VF, Ventricular Tachycardia/Ventricular Fibrillation.

An overview of both analyses is depicted in Figure 1.

### Holter

Holters recordings were obtained with a standard 125Hz 3 lead Holter registration system (SEER Light Holter Recorder, GE healthcare, UK). A trained nurse attached all devices and placed leads at locations V1, V3, and V5. All registrations were done outside the hospital and patients were instructed to follow their normal routine. RR, QT (measured both until the peak (QTp) and end (QTe) of T-wave) and QTc (corrected with the Bazett's Formula) interval were measured every 15 s. Hourly averages were calculated using the manufacturers software (MARS version 7.2, GE Healthcare, UK). Based on the hourly averages, the best-fitting cosine curves with a period of 24-h were calculated for all parameters using a non-linear regression model (R statistics Version 3.0, R Development Core Team, New Zealand).

**Figure 2:**
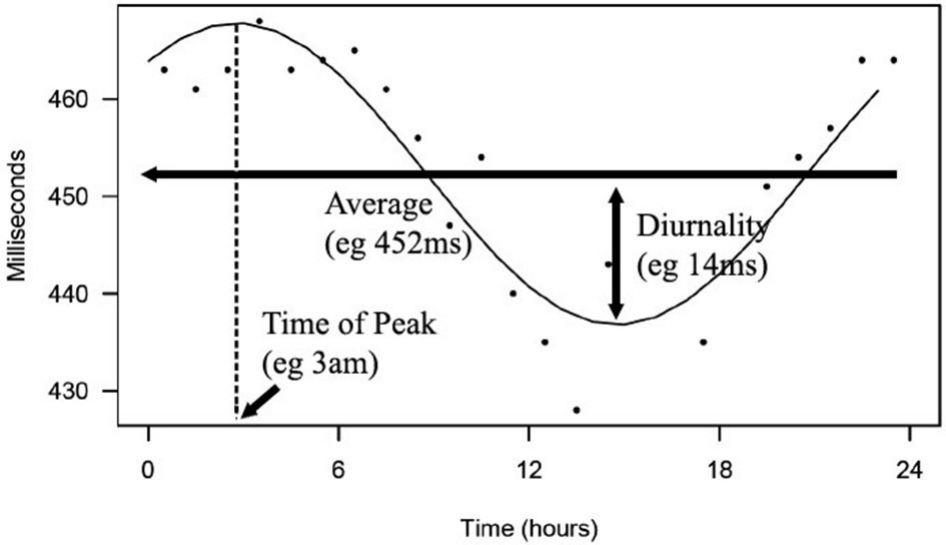
Depicted is a 24-h rhythm in QT of one of the patients. From hourly averages, an optimal fitting cosine curve with a period of 24-h was calculated. 24-h average, peak time, and diurnality were derived from this curve.

From this cosine curve, 24-h averages, peak time, and diurnality were derived. We defined diurnality as the amplitude of the cosine curve, or half of the difference between peak and trough of the 24-h cosine curve. QT analysis and a visualization of diurnality of one of the patients are depicted in Figure 2.

### Statistical analysis

Data is presented as averages ± standard deviation. Pearson's correlation coefficient was used to compare variables. Levene's test was used to check equality of variances. When homoscedastic, one- or two-way analysis of variance (ANOVA) was conducted to compare groups. If not, the Kruskal-Wallis test was performed. In analyses with multiple groups, Bonferroni *post-hoc* analysis was used to compare subjects with controls. Pearson's chi-squared test was used to compare categorical data. *P* < 0.05 were considered statistically significant. Predictive power of parameters that were significantly different between groups was quantified using area under curves (AUC) of the receiver-operator characteristics.

## Results

### 24-h rhythms in repolarization and ventricular arrhythmias

#### Patient collection

Thirty-nine patients with systolic heart failure and a history of myocardial infarction were included (see Table 1). Baseline characteristics of all patients are depicted in Table 2. The only significant difference between patients with and without arrhythmias was the prevalence of Diabetes Mellitus (DM). Our main parameters, QTd and QTcd however, did not differ between patients with and without DM (QTd 20 ± 19 vs. 23 ± 11 ms, *p* = 0.90; QTcd 13 ± 20 vs. 13 ± 7 ms *p* = 0.99, respectively).

**Table 2:** Patient characteristics at time of Holter.

**Baseline Characteristics**	**VT/VF− *n* = 25**	**VT/VF+ *n* = 14**	**Significance**
Age (years)	51 ± 16	55 ± 10	ns
Gender (% male)	80	79	ns
Ejection Fraction (%)	29 ± 5	26 ± 5	ns
LVEDD (cm)	6.3 ± 0.9	6.2 ± 1.2	ns
LVESD (cm)	5.2 ± 1.0	5.3 ± 1.0	ns
EDV (ml)	181 ± 74	198 ± 91	ns
ESV (ml)	120 ± 42	150 ± 73	ns
SV (ml)	60 ± 29	51 ± 24	ns
β-blocker (used in all patients, converted to mg metoprolol)	113 ± 102	98 ± 75	ns
GRF (ml/min)	51 ± 16	55 ± 10	ns
DM (%)	44	7	*p* = 0.017
NYHA Classification	2.1 ± 0.8	1.9 ± 0.5	ns

*DM, Diabetes Mellitus; EDV, End Diastolic Volume; ESV, End Systolic Volume; GFR, Glomerular Filtration Rate; LVEDD, Left Ventricular End Diastolic Diameter; LVESD, Left Ventricular End Systolic Diameter; ns, not significant; SV, Stroke Volume; VT/VF, Ventricular Tachycardia/Ventricular Fibrillation*.

#### Accurate automatic 24-h measurements

Previous studies showed that automatic measurements of the QT interval can be challenging (Tyl et al., 2011). To assure the accuracy of the automated measurements in this current study, we performed several validations that are specified in the supplementary data. (Supplementary Figures 1–3).

#### Ventricular arrhythmias are associated with high QTd/QTcd

**Figure 3:**
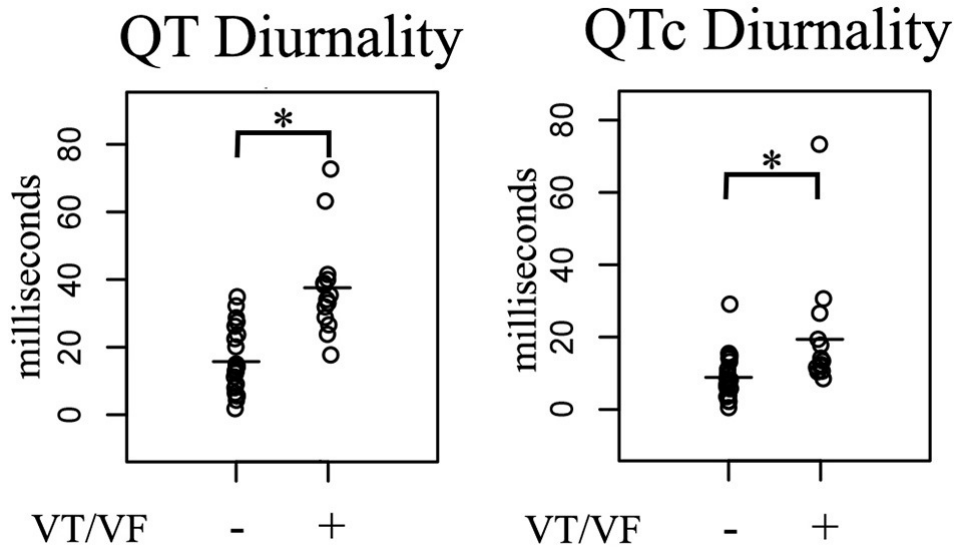
Comparison of 24-h Holter parameters in post-infarct systolic heart failure patients subdivided in two groups based on history of ventricular arrhythmias. In arrhythmia patients, QT(c) diurnality (QTd and QTcd, respectively) were two-fold higher compared to non-arrhythmia controls. VT/VF, Ventricular Tachycardia/Ventricular Fibrillation. ^*^p < 0.05 vs. no arrhythmia.

24-h rhythms in RR and QT(c) interval duration were compared between patients with and without history of ventricular arrhythmias. History of ventricular arrhythmias was associated with a two-fold increase of QTd/QTcd as compared to CHF VT- patients (QTd 38 ± 15 vs. 16 ± 9 ms *p* = 0.001 and QTcd 19 ± 17 vs. 9 ± 6 ms, respectively, *p* = 0.003). In addition to these large differences, overlap of QTd values between the two groups was low: the lowest QTd of patients with ventricular arrhythmias was 18 ms, whereas approximately two-thirds (17/25) of the VT- patients had a QTd below this value (Figure 3 and Table 3). This resulted in a high discriminative power (Area under curve (AUC) of receiver operating characteristic (ROC) curve QTd and QTcd, 0.932 ± 0.038 and 0.818 ± 0.068, respectively).

**Table 3:** Holter parameters of systolic heart failure patients with and without history of VT/VF.

**Baseline characteristics**	**VT/VF− *n* = 25**	**VT/VF+ *n* = 14**	**Significance**
RR Peak Time (h)	4.5 ± 3.9	3.8 ± 2.2	ns
QT Peak Time (h)	5.4 ± 6.3	4.4 ± 2.7	ns
QTc Peak Time (h)	8.8 ± 7.2	9.6 ± 6.1	ns
RR Average (ms)	861 ± 107	895 ± 133	ns
QT Average (ms)	425 ± 29	428 ± 33	ns
QTc Average (ms)	460 ± 25	455 ± 20	ns
RR Diurnality (ms)	70 ± 41	111 ± 43	ns
QT Diurnality (ms)	16 ± 9	38 ± 15	*p = 0.001*
QTc Diurnality (ms)	9 ± 6	19 ± 17	*p* = 0.003
QRS Duration (ms)	119 ± 24	112 ± 31	ns
HRV (SDNN)	114 ± 38	107 ± 35	ns

*HRV, Heart Rate Variability; ns, not significant; VT/VF, Ventricular Tachycardia/Ventricular Fibrillation*.

### 24-h rhythms in repolarization and hERG channel functionality

Cardiovascular disease is associated with, in general, tempered or disrupted 24-h rhythms (Durgan and Young, 2010; Hermida et al., 2011; Jeyaraj et al., 2012). Schroder et al. published a study which showed that normal expression of KCNH2, a gene encoding the hERG (human Ether-à-go-go-Related Gene, K_v_11.1) protein, has a 24-h rhythm in mice (Schroder et al., 2015). hERG is the α-subunit of the delayed rectifier current (I_Kr_) channel, a potassium channel which mediates repolarization duration of the action potential (Sanguinetti et al., 1995). Schroder et al showed that the 24-h rhythm in hERG channel expression suppresses a rhythm in repolarization duration under physiological conditions. Consequently, when rhythmicity in hERG channel expression was disrupted, the 24-h rhythm in repolarization increased.

Based on this observation, we hypothesized that in humans, a rhythm in ion channel functioning, such as the hERG channel, could also be of importance to control a 24-h rhythm in repolarization. If so, alike in mice, this could explain why an increase of QTd (and not a decrease) is associated with ventricular arrhythmias. To test this hypothesis, in a second retrospective study, we investigated whether depression of hERG channel functionality in patients resulted in an increase of QTd by comparing QTd of patients with a proven hERG channel dysfunction (LQTS2) or those in which depressed hERG channel functionality is anticipated (Sotalol treated), to controls.

#### Patient collection

Thirty-four patients were included. Twenty-two Holters were recorded during control conditions (no LQTS2 or Sotalol use), 16 during a regimen of Sotalol treatment and five in patients with LQTS2. In nine patients, two separate Holters were performed, one Holter before administration of Sotalol and one Holter during Sotalol usage. Time between those two Holters was 12 ± 11 months. As such 43 Holters were available in total. Age and gender were similar between control patients and Sotalol users (59 ± 10 vs. 62 ± 12 years and 59 vs. 63% male, respectively). Four out of five patients diagnosed with LQTS2 were female, these patients were 49 ± 6 years old at the time of their Holter and all used beta-blockers. LQTS2 mutations were c.2959_2960delCT (2x), c.260T > C, c.2887C > A, and c.2354G > T. LVEF was similar in all groups (57 ± 5% vs. 57 ± 6% vs. 58 ± 4%).

#### Sotalol usage is associated with high QTd/QTcd

**Figure 4:**
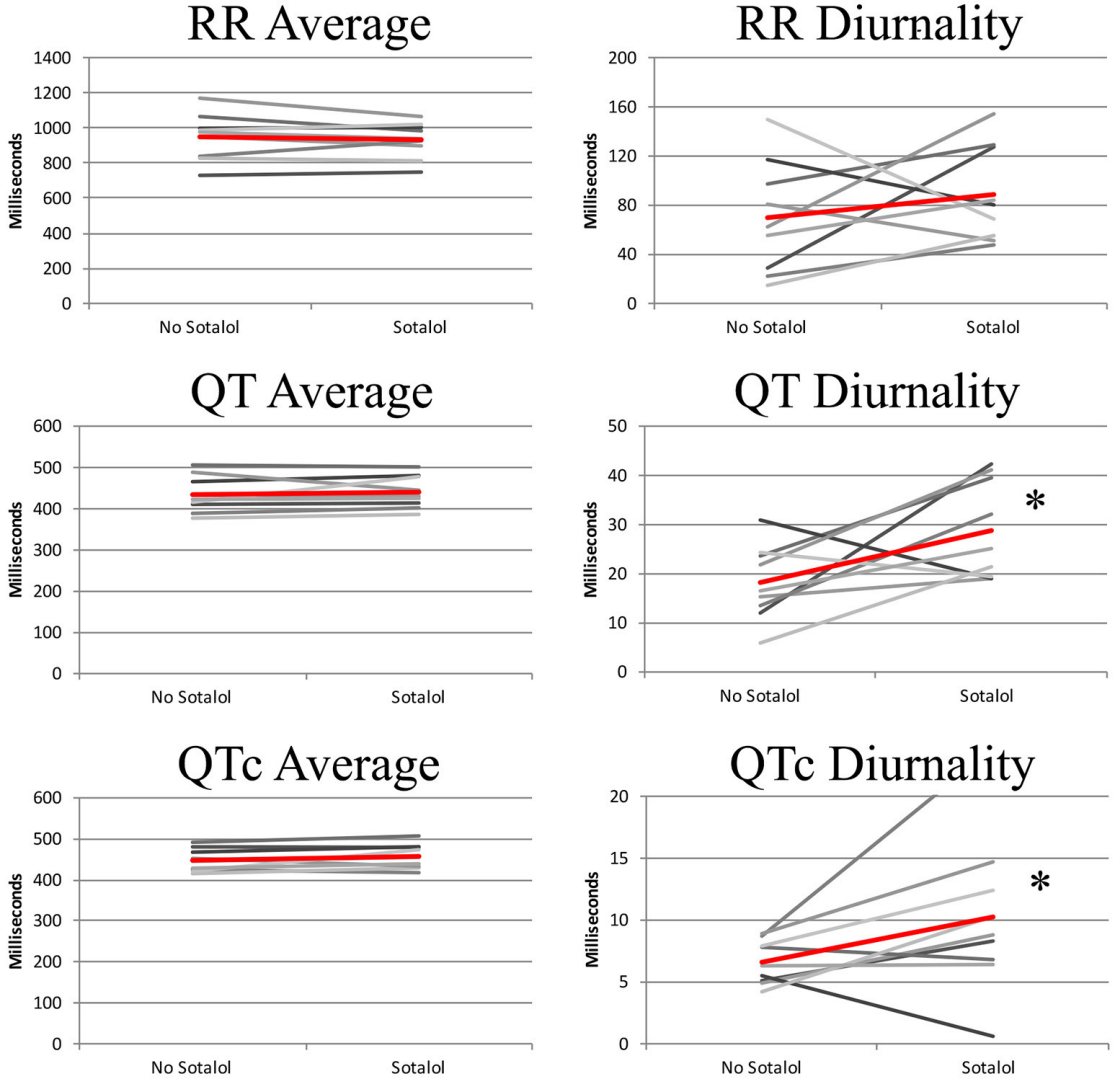
24-h QT and QTc diurnality (QTd and QTcd, respectively) is higher in patients using Sotalol compared to the same patients without anti-arrhythmic drugs. Red line indicates average of all subjects. ^*^P < 0.05 for difference between no Sotalol and Sotalol.

In the nine patients in whom two Holter recordings were available (with and without Sotalol), 24-h averages, QT diurnality, and peak time of RR, QT, and QTc were analyzed. Peak times, 24 h averages, and RR diurnality did not differ significantly between the two Holters (Table 4). Remarkably, QTd and QTcd increased with Sotalol use (10 ± 13 and 4 ± 6 ms increase, *p* = 0.02 and 0.04, respectively). Data are depicted in Figure 4.

**Table 4:** Holter parameters of patients with Holter with and without Sotalol.

**Baseline characteristics**	**Sotalol− *n* = 9**	**Sotalol+ *n* = 9**	**Significance**
RR Peak Time (h)	2.8 ± 2.4	2.9 ± 2.0	ns
QT Peak Time (h)	2.6 ± 2.4	2.6 ± 1.9	ns
QTc Peak Time (h)	3.8 ± 5.7	3.5 ± 7.0	ns
RR Average (ms)	948 ± 133	932 ± 101	ns
QT Average (ms)	434 ± 44	440 ± 39	ns
QTc Average (ms)	447 ± 28	457 ± 30	ns
RR Diurnality (ms)	70 ± 46	89 ± 39	ns
QT Diurnality (ms)	18 ± 8	29 ± 10	*p* = 0.02
QTc Diurnality (ms)	7 ± 2	10 ± 6	*p* = 0.04

*ns: not significant*.

Sotalol, especially at lower doses, does not lead to full hERG channel dysfunction (Hohnloser and Woosley, 1994). To further explore the relation between repolarization and Sotalol, we therefore compared daily Sotalol dosage to QTd and QTcd. For QTd, no relation was found (*R*^2^ = 0.02, *p* = 0.59), but as expected, longer QTcd was associated with higher doses of Sotalol (*R*^2^ = 0.35 *p* = 0.02). These findings correspond to the known dose-dependent pro-arrhythmic effects of Sotalol (Hohnloser, 1997). More importantly, patients with the lowest daily Sotalol dosage (80 mg) all had low (<10 ms) QTcd values (Supplemental Figure 1). Previous studies revealed that low doses of Sotalol have class I (beta-adrenergic) anti-arrhythmic effects, but no effects on repolarization (class III), including pro-arrhythmic side effects (Hohnloser, 1997; Singh, 1999). We therefore excluded patients with a daily dose of 80 mg Sotalol from further analysis. Mean dose of the remaining subjects was 196 mg (range 120–320 mg).

#### hERG channel dysfunction is associated with QTd and QTcd

**Figure 5:**
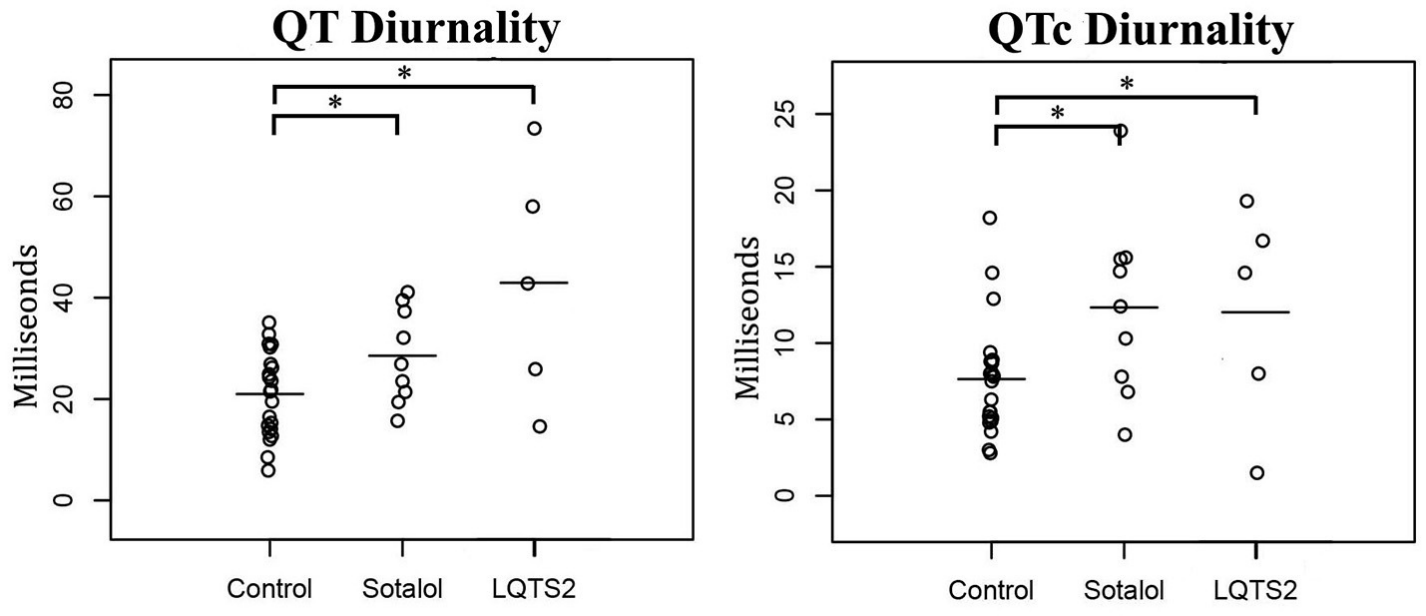
Comparison of 24-h Holter parameters between controls, Sotalol users, and LQTS2 patients. QT and QTc diurnality are increased in Sotalol users and LQTS2 patients. ^*^indicates *p* < 0.05 compared to controls.

As compared to controls, QTd and QTcd were increased in both Sotalol users and LQTS2 patients (QTd 21 ± 8 vs. 29 ± 9 and 43 ± 24 ms, *p* = 0.031 and *p* = 0.001 QTcd 8 ± 4 vs. 12 ± 6 and 12 ± 7 ms, *p* = 0.027 and *p* = 0.049 for controls vs. Sotalol, and LQTS2 resp.). Compared to those controls, 24-h QT and QTc averages were significantly higher in LQTS2 patients (QT 421 ± 37 ms vs. 474 ± 18, *p* = 0.010 and QTc 448 ± 20 vs. 494 ± 33 ms, *p* = 0.002 resp.), but no other significant 24-h differences were found between Sotalol / LQTS2 vs. controls (Data in Table 5 and Figure 5). These data confirm our hypothesis that hERG channel dysfunction is associated with QTd and QTcd and illustrates that in humans, an increase in rhythmicity (instead of the usually seen decrease) can be associated with disease.

**Table 5:** Holter parameters of Sotalol users, LQTS2 patients and control patients.

**Baseline characteristics**	**Controls (*n* = 22)**	**Sotalol (*n* = 9)**	**LQTS2 (*n* = 5)**	**Significance (controls vs. sotalol and LQTS2 resp.)**
RR Average (ms)	897 ± 144	954 ± 73	936 ± 104	Both ns
QT Average (ms)	421 ± 37	442 ± 37	474 ± 18	ns, *p* = 0.010
QTc Average (ms)	448 ± 20	454 ± 29	494 ± 33	ns, *p* = 0.002
RR Diurnality (ms)	83 ± 36	89 ± 47	119 ± 66	Both ns
QT Diurnality (ms)	21 ± 8	29 ± 9	43 ± 24	*p* = 0.031 and *p* = 0.001
QTc Diurnality (ms)	8 ± 4	12 ± 6	12 ± 7	*p* = 0.027 and *p* = 0.049

*LQTS2, Patients with proven mutation in hERG channel; ns, not significant*.

## Discussion

In this study, we investigated 24-h rhythms in repolarization and introduced the new clinical parameters QT and QTc diurnality (QTd and QTcd, respectively). In a retrospective study, we showed that QTd and QTcd are associated with ventricular arrhythmias in post-infarct patients with a LVEF <35%. We found that ventricular arrhythmias are associated with a high QTd and QTcd. About 2/3 of patients without arrhythmias showed values of QTd lower than the value of the arrhythmia-positive patient with the lowest QTd value in that group. In an attempt to deduce a potential causative explanation for these observations, we performed a second retrospective study and revealed that (high) QTd and QTcd seem associated with hERG channel dysfunction.

Susceptibility for repolarization related ventricular tachyarrhythmias is often related to an inherited and/or acquired reduced repolarization reserve, which regularly includes a downregulation of the hERG protein and function. To quantify repolarization reserve, QT length is not optimal and other techniques have been applied in the scientific community: (1) exaggerated increase in QT after a hERG blocking drug (Kaab et al., 2003), (2) parameters of temporal repolarization dispersion quantified as temporal QTVI/N (Berger et al., 1997) and STV (Hinterseer et al., 2008), (3) alterations in T wave morphology after the administration of a hERG blocking drug (Couderc et al., 2011). So far however, these parameters are not widely used in daily practice.

Some previous publications confirmed the relation between 24-h rhythm and repolarization parameters. QTVI and QTVN for example, two short-term repolarization parameters, showed a diurnal rhythm (Dobson et al., 2009). In addition, Van der Berg et al. showed that at night, maximum ventricular repolarization duration corresponds to an increased incidence of SCD in LQT3-Brugada patients (van den Berg et al., 2006). To our knowledge however, the current study is the first to study diurnality (amplitude) of repolarization and identifies this parameter as potentially discriminating/predictive for ventricular arrhythmias in humans.

In the current study, we propose that QTd and QTcd are associated with hERG channel dysfunction. Expression profiles of several cardiac ion channels are disrupted in heart failure patients (Sugiyama et al., 2011). We hesitate however, that dysfunction of the hERG channels fully explains the increase in QTc/QTcd and occurrence of ventricular arrhythmias. hERG channel disruption for example, is often associated with other electrophysiological changes such as an increase in (average) QTc or HRV. The patients with reduced LVEF in the current study that faced arrhythmic events, however, do not show these changes. In addition, differences between arrhythmia and non-arrhythmia patients are larger for QTd than for QTcd, suggesting that other factors such as changes in autonomic drive may have contributed to the differences in QTd and QTcd as well. Also in the patients that used Sotalol for treatment of AF, QT, and QTc are not prolonged though in these patients we anticipate only a mild degree of hERG block to avoid triggering of ventricular arrhythmias. In line with that, QTd shows an increased value though to a much lower extent when compared to the patients with LQTS2 or those with a reduced EF. On the other hand, in the small group of LQTS2 patients that do have a proven hERG dysfunction and a history of VT but without a low EF, QTd value was even higher as compared to the QTd of the group with low EF and, in this case also QT and QTc were significantly prolonged.

Correct 24-h measurement of the QT interval is challenging. Not only is the QT interval under influence of heart rate and has automatic measurement proven difficult, positional changes of heart within the thorax and fluctuations in drug plasma levels during the day complicate accurate measurement for longer periods even further. To minimize the risk of inaccurate measurements, we used several strategies. First, automated measurements were done with validated, commercially used software (MARS version 7.2, GE Healthcare, UK) and we checked whether measurements were accurate (Supplemental Figures 2–4).

Secondly, to prevent any interaction of fluctuating drug plasma levels we excluded all patients with anti-arrhythmic drugs despite those that used slow-releasing beta-blockers, which beta-adrenergic effects have been reported to be relatively constant (Sandberg et al., 1988). In the subgroup of Sotalol patients, we analyzed the effects of fluctuating plasma levels on QTd because of the relatively short half-life (±12 h). Peak times of the 24-h QT rhythm in Sotalol users were similar to controls. In addition, ECG changes reach a maximum at 1.5 h after Sotalol administration (Singh, 1999). The single QT peak in the middle of the night that is observed in Sotalol users, is therefore unlikely to be caused by a drug regimen of two intakes in the morning and evening.

Third, because repolarization duration depends on heart rate, we considered an effect of 24-h heart rate variation on QTd. To measure heart-rate independent repolarization values, several formulas were developed, such as Bazett's Formula, Fridericia's Formula, and individually optimized curvature corrections. Previous studies showed that 24-h rhythms in QTc depend on the method used (Malik et al., 2008). In our study, we used Bazett's formula. We purposely did not correct for 24-h QT/RR hyperesthesis since this filters 24-h rhythms, but other methods such as Frederica's Formula or QT/RR hyperesthesis taken at a specific time point might have yielded different results. In addition, differences in heart rate, for example caused by beta-adrenergic effects of Sotalol, have previously shown to affect Sotalol activity (Hayward and Taggart, 1986). The QTd data of Sotalol users vs. controls however, shows similar RR averages and RR diurnality levels in combination with significant differences in QTd, confirming that the differences in QTd found are independent of heart rate.

Finally, there is a risk that the position of the heart in the thorax changes throughout the day. However, patient characteristics of those positive for ventricular arrhythmia patients and controls were similar (age, gender, cardiac function, severity of symptoms). Even though 24-h variation in cardiac position may have influenced QTd, we could not define any reason why this would be different in arrhythmia positive patients compared to those who were devoid of arrhythmias.

### Clinical implications

In the post-infarct patient with a LVEF <35%, differences in QTd between patients with and without arrhythmias were not only statistically significant, but also large: 2/3 of the patients without arrhythmias have a lower QTd than the arrhythmia-positive patient with the lowest QTd value. According to current guidelines, all patients with an LVEF <35% receive an ICD, but only a minority will get a ventricular arrhythmia and benefit from this ICD. 80% of those patients receive an ICD without any benefit but with the risk of complications such as lead revisions and inappropriate shocks. In combination with the high costs of an ICD implantation, there is an urgent clinical need for a better risk stratification in this patient group. The large difference in QTd values and the small overlap between patients with and without history of ventricular arrhythmias, make QTd a promising candidate to follow up as a new and additive arrhythmia predictor in patients at risk.

### Study limitations

Because our study was observational, we are not able to confirm a direct causal relationship between hERG channel dysfunction, QTd, and development of ventricular arrhythmias. Secondly, the retrospective study setup and relatively small patient groups do not allow predictive conclusions. To achieve in that, further prospective studies in large patient cohorts will be required in order to reproduce our findings, evaluate the definite predictive value of QTd, and to the define cut-off values for QTd that discriminate between being at risk or not.

## Ethics statement

This study was carried out in accordance with the recommendations of Medical Ethical Committee of the UMC Utrecht with written informed consent from all subjects. All subjects gave written informed consent in accordance with the Declaration of Helsinki. The protocol was approved by the Medical Ethical Committee of the UMC Utrecht.

## Author contributions

All authors made substantial contributions to the conception of the work. BD did the analysis and interpretation of data for the work. BD drafted the work, all others revised it critically for important intellectual content, approved with the final version and agree to be accountable for all aspects of the work in ensuring that questions related to the accuracy or integrity of any part of the work are appropriately investigated and resolved.

### Conflict of interest statement

The authors declare that the research was conducted in the absence of any commercial or financial relationships that could be construed as a potential conflict of interest.

## Supplementary material

Supplemental Figure 1 QTc diurnality correlates with Sotalol dosage. More specifically, patients receiving low Sotalol amounts (80 mg/day, indicated in red) had a relatively low QTc diurnality.

Supplemental Figure 2 Comparison of manual and automated 24-h Holter parameters. RR interval, Time between 2 QRS complexes; QTe interval, QT time measured until the end of the T-wave.

Supplemental Figure 3 Comparison of automated Holter calculations. **Left:** QT interval from Q-wave to top of T-wave is compared to QT interval from Q-wave to the end of the T-wave. **Right:** 24-h parameters from lead V5 are compared to lead V3. QTe, QT interval measured until the end of T-wave; QTp, QT interval measured until the peak of the T-wave.

Supplemental Figure 4 **Left:** 24-h QT diurnality correlates with 24-h range. Red dot represents one of the outliers and is further analyzed in right side of figure. **Right:** 24-h rhythm in QT interval of example outlier. QT diurnality was calculated as difference between average and maximum of optimal cosine curve. QT range was calculated as difference between two maximal and minimal dots (depicted in red). The relatively high range in the example is caused by one very low value (red dot at bottom).

## Supplemental data

### Accuracy of automatic 24-h measurements

To check the accuracy of the automated measurements in this study, we compared manual and automated calculations of the 24-h rhythm in QT interval. Although, absolute values slightly differed, the correlation between manual and automated measurements was high (*R*^2^ = 0.97, *p* < 0.001, Supplemental Figure 2).

To further validate the automated measurements, we compared different QT measurement options. QTend values (QT interval from onset of the QRS-complex to the end of the T-wave; used in the current study) were compared to QTpeak values (until the top of the T-wave). QTend values were ±80 ms larger than QTpeak values and were strongly correlated (*R*^2^ = 0.99 *p* < 0.001, Supplemental Figure 3). Next, QT measurements of different leads were compared. Lead V5 (used in the analyses) and V3, and V5 and V1 have slightly different (±15 ms) absolute values but correlate very well (*R*^2^ = 0.99, *p* < 0.001 and *R*^2^ = 0.97 *p* < 0.001, respectively, V5 vs. V3 in Supplemental Figure 3).

Finally, we compared cosine curve analysis to other methods of measuring 24-h parameters. 24-h averages derived from the cosine curve matched the average of the 24-hourly averages (*R*^2^ = 0.99, *p* < 0.001). Peak times derived from the cosine curve corresponded to the time point of the maximum hourly average (*R*^2^ = 0.87, *p* < 0.001) and diurnality correlated to the difference between the two maximal and minimal hourly averages (range, *R*^2^ = 0.83, *p* < 0.001). Importantly, differences between QTd or QTcd, and range were often caused by 1 or 2 outliers (Supplemental Figure 4) that affected range, but not diurnality.

## References

[B1] Bardy G. H. Lee K. L. Mark D. B. Poole J. E. Packer D. L. Boineau R. (2005). Amiodarone or an implantable cardioverter-defibrillator for congestive heart failure. N. Engl. J. Med. 352, 225–237. 10.1056/NEJMoa043399 15659722

[B2] Berger R. D. Kasper E. K. Baughman K. L. Marban E. Calkins H. Tomaselli G. F. (1997). Beat-to-beat QT interval variability: novel evidence for repolarization lability in ischemic and nonischemic dilated cardiomyopathy. Circulation 96, 1557–1565. 10.1161/01.CIR.96.5.1557 9315547

[B3] Couderc J. P. Xia X. Peterson D. R. McNitt S. Zhao H. Polonsky S.. (2011). T-wave morphology abnormalities in benign, potent, and arrhythmogenic I(kr) inhibition. Heart Rhythm 8, 1036–1043. 10.1016/j.hrthm.2011.02.005 21315844 PMC3145317

[B4] Dobson C. P. La Rovere M. T. Olsen C. Berardinangeli M. Veniani M. Midi P.. (2009). 24-hour QT variability in heart failure. J. Electrocardiol. 42, 500–504. 10.1016/j.jelectrocard.2009.06.021 19647268

[B5] Durgan D. J. Young M. E. (2010). The cardiomyocyte circadian clock: emerging roles in health and disease. Circ. Res. 106, 647–658. 10.1161/CIRCRESAHA.109.209957 20203314 PMC3223121

[B6] Hayward R. P. Taggart P. (1986). Effect of sotalol on human atrial action potential duration and refractoriness: cycle length dependency of class III activity. Cardiovasc. Res. 20, 100–107. 10.1093/cvr/20.2.100 3708643

[B7] Hermida R. C. Ayala D. E. Mojon A. Fernandez J. R. (2011). Decreasing sleep-time blood pressure determined by ambulatory monitoring reduces cardiovascular risk. J. Am. Coll. Cardiol. 58, 1165–1173. 10.1016/j.jacc.2011.04.043 21884956

[B8] Hinterseer M. Thomsen M. B. Beckmann B. M. Pfeufer A. Schimpf R. Wichmann H. E.. (2008). Beat-to-beat variability of QT intervals is increased in patients with drug-induced long-QT syndrome: a case control pilot study. Eur. Heart J. 29, 185–190. 10.1093/eurheartj/ehm586 18156612

[B9] Hohnloser S. H. (1997). Proarrhythmia with class III antiarrhythmic drugs: types, risks, and management. Am. J. Cardiol. 80, 82G–89G. 10.1016/S0002-9149(97)00717-0 9354415

[B10] Hohnloser S. H. Woosley R. L. (1994). Sotalol. N. Engl. J. Med. 331, 31–38. 10.1056/NEJM199407073310108 8202100

[B11] ICH S7B guidelines. (2015). ICH Harmonised Tripartite Guideline. The Non-Clinical Evaluation of the Potential for Delayed Ventricular Repolarization (QT Interval Prolongation) by Human Pharmaceuticals. S7B. 2005. Available online at: http://ichguideline.weebly.com/uploads/2/6/2/1/26210522/s7b_step4.pdf Accessed date june 2017.

[B12] Jeyaraj D. Haldar S. M. Wan X. McCauley M. D. Ripperger J. A. Hu K.. (2012). Circadian rhythms govern cardiac repolarization and arrhythmogenesis. Nature 483, 96–99. 10.1038/nature10852 22367544 PMC3297978

[B13] Kaab S. Hinterseer M. Nabauer M. Steinbeck G. (2003). Sotalol testing unmasks altered repolarization in patients with suspected acquired long-QT-syndrome–a case-control pilot study using i.v. sotalol. Eur. Heart J. 24, 649–657. 10.1016/S0195-668X(02)00806-0 12657223

[B14] Malik M. Hnatkova K. Schmidt A. Smetana P. (2008). Accurately measured and properly heart-rate corrected QTc intervals show little daytime variability. Heart Rhythm 5, 1424–1431. 10.1016/j.hrthm.2008.07.023 18929329

[B15] Moss A. J. Zareba W. Hall W. J. Klein H. Wilber D. J. Cannom D. S.. (2002). Prophylactic implantation of a defibrillator in patients with myocardial infarction and reduced ejection fraction. N. Engl. J. Med. 346, 877–883. 10.1056/NEJMoa013474 11907286

[B16] Nakagawa M. Iwao T. Ishida S. Yonemochi H. Fujino T. Saikawa T.. (1998). Circadian rhythm of the signal averaged electrocardiogram and its relation to heart rate variability in healthy subjects. Heart 79, 493–496. 10.1136/hrt.79.5.493 9659198 PMC1728677

[B17] Niemeijer M. N. van den Berg M. E. Leening M. J. Hofman A. Franco O. H. Deckers J. W.. (2015). Declining incidence of sudden cardiac death from 1990-2010 in a general middle-aged and elderly population: The Rotterdam Study. Heart Rhythm 12, 123–129. 10.1016/j.hrthm.2014.09.054 25277989

[B18] Sandberg A. Blomqvist I. Jonsson U. E. Lundborg P. (1988). Pharmacokinetic and pharmacodynamic properties of a new controlled-release formulation of metoprolol: a comparison with conventional tablets. Eur. J. Clin. Pharmacol. 33(Suppl), S9–S14. 10.1007/BF00578406 3371395

[B19] Sanguinetti M. C. Jiang C. Curran M. E. Keating M. T. (1995). A mechanistic link between an inherited and an acquired cardiac arrhythmia: HERG encodes the IKr potassium channel. Cell 81, 299–307. 10.1016/0092-8674(95)90340-2 7736582

[B20] Schroder E. A. Burgess D. E. Zhang X. Lefta M. Smith J. L. Patwardhan A.. (2015). The cardiomyocyte molecular clock regulates the circadian expression of Kcnh2 and contributes to ventricular repolarization. Heart Rhythm 12, 1306–1314. 10.1016/j.hrthm.2015.02.019 25701773 PMC4541807

[B21] Seegers J. Vos M. A. Flevari P. Willems R. Sohns C. Vollmann D.. (2012). Rationale, objectives, and design of the EUTrigTreat clinical study: a prospective observational study for arrhythmia risk stratification and assessment of interrelationships among repolarization markers and genotype. Europace 14, 416–422. 10.1093/europace/eur352 22117037 PMC3283222

[B22] Singh B. N. (1999). Sotalol: current status and expanding indications. J. Cardiovasc. Pharmacol. Ther. 4, 49–65. 10.1177/107424849900400108 10684524

[B23] Sugiyama H. Nakamura K. Morita H. Akagi S. Tani Y. Katayama Y.. (2011). Circulating KCNH2 current-activating factor in patients with heart failure and ventricular tachyarrhythmia. PLoS ONE 6:e19897. 10.1371/journal.pone.0019897 21625547 PMC3098251

[B24] Szwejkowski B. R. Wright G. A. Connelly D. T. Gardner R. S. (2015). When to consider an implantable cardioverter defibrillator following myocardial infarction? Heart 101, 1996–2000. 10.1136/heartjnl-2015-307788 26526420

[B25] Tyl B. Azzam S. Blanco N. Wheeler W. (2011). Improvement and limitation of the reliability of automated QT measurement by recent algorithms. J. Electrocardiol. 44, 320–325. 10.1016/j.jelectrocard.2010.11.006 21163494

[B26] van den Berg M. P. Haaksma J. Veeger N. J. Wilde A. A. (2006). Diurnal variation of ventricular repolarization in a large family with LQT3-Brugada syndrome characterized by nocturnal sudden death. Heart Rhythm 3, 290–295. 10.1016/j.hrthm.2005.11.023 16500301

[B27] Zipes D. P. Camm A. J. Borggrefe M. Buxton A. E. Chaitman B. Fromer M.. (2006). ACC/AHA/ESC 2006 guidelines for management of patients with ventricular arrhythmias and the prevention of sudden cardiac death–executive summary: a report of the American College of Cardiology/American Heart Association Task Force and the European Society of Cardiology Committee for Practice Guidelines (Writing Committee to Develop Guidelines for Management of Patients with Ventricular Arrhythmias and the Prevention of Sudden Cardiac Death) Developed in collaboration with the European Heart Rhythm Association and the Heart Rhythm Society. Eur. Heart J. 27, 2099–2140. 10.1093/eurheartj/ehl199 16923744

