# Supplementary figures and images for: Analysis of 24-h Rhythm in Ventricular Repolarization Identifies QT Diurnality As a Novel Clinical Parameter Associated with Previous Ventricular Arrhythmias in Heart Failure Patients
Source: Front Physiol. 2017 Aug 15;8:590. doi: 10.3389/fphys.2017.00590 (PMC5559512; doi:10.3389/fphys.2017.00590)

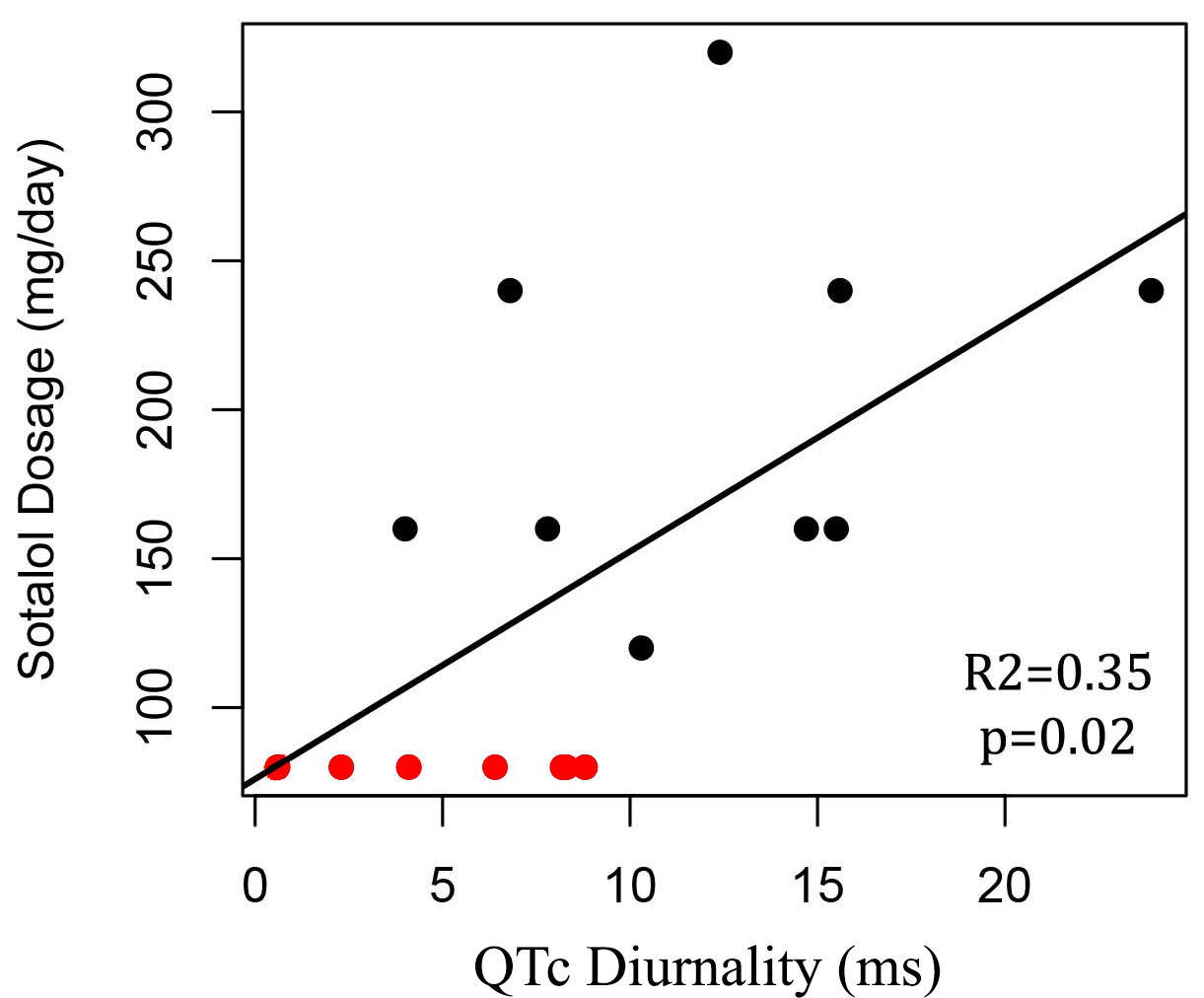

Supplement: Supplemental Figure 1 — QTc diurnality correlates with Sotalol dosage. More specifically, patients receiving low Sotalol amounts (80 mg/day, indicated in red) had a relatively low QTc diurnality. [file Image1.JPEG]

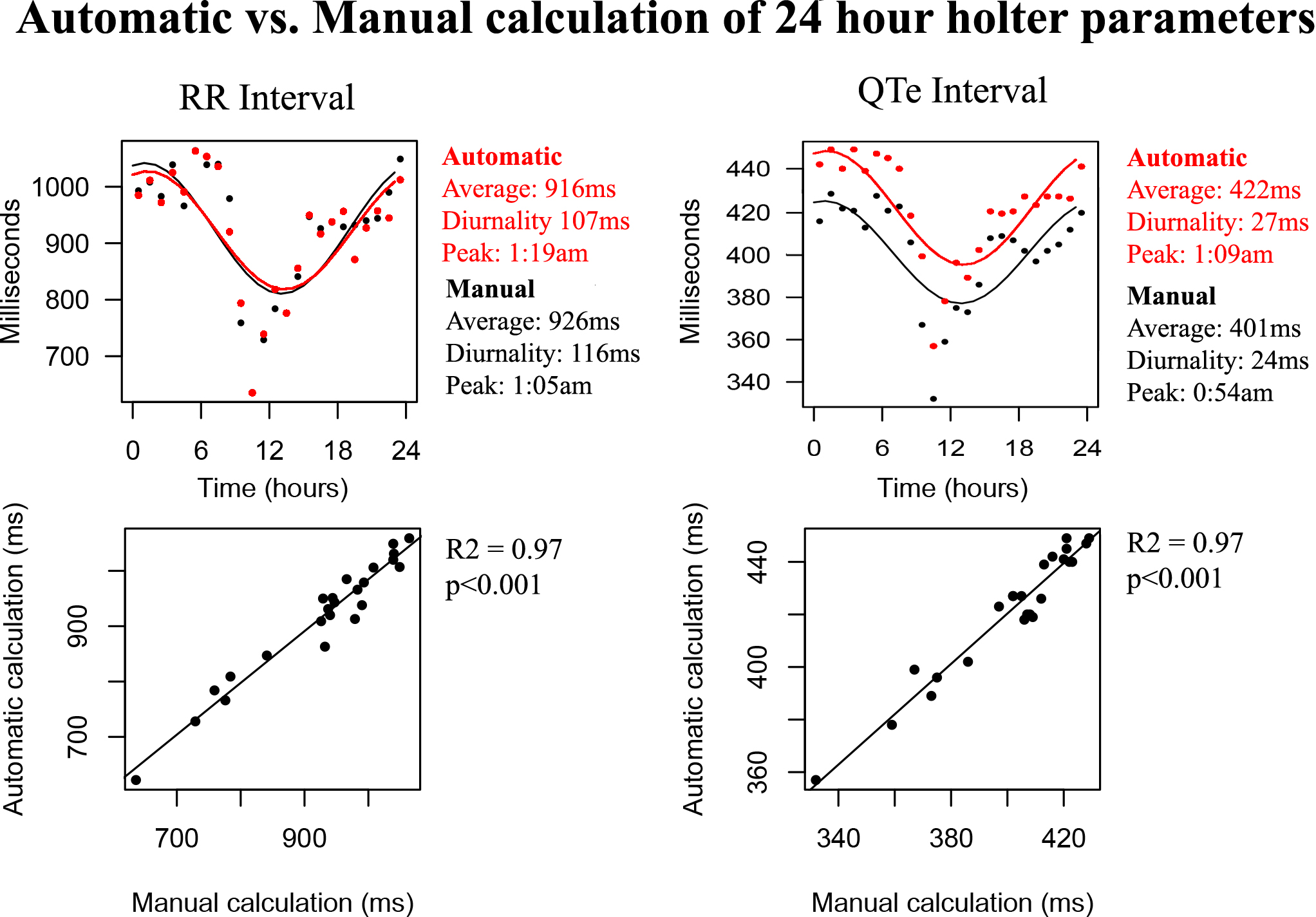

Supplement: Supplemental Figure 2 — Comparison of manual and automated 24-h Holter parameters. RR interval, Time between 2 QRS complexes; QTe interval, QT time measured until the end of the T-wave. [file Image2.JPEG]

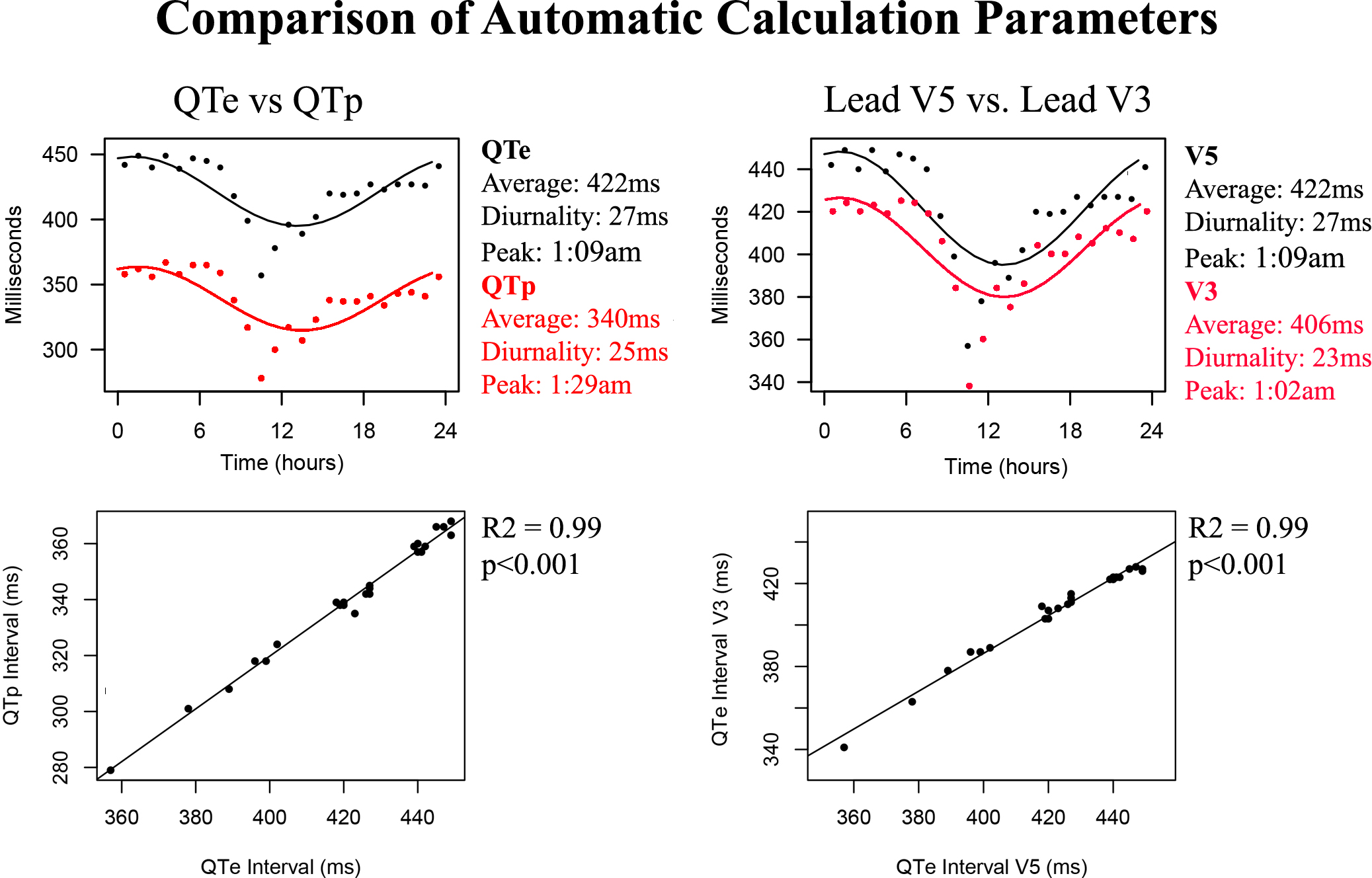

Supplement: Supplemental Figure 3 — Comparison of automated Holter calculations. Left: QT interval from Q-wave to top of T-wave is compared to QT interval from Q-wave to the end of the T-wave. Right: 24-h parameters from lead V5 are compared to lead V3. QTe, QT interval measured until the end of T-wave; QTp, QT interval measured until the peak of the T-wave. [file Image3.JPEG]

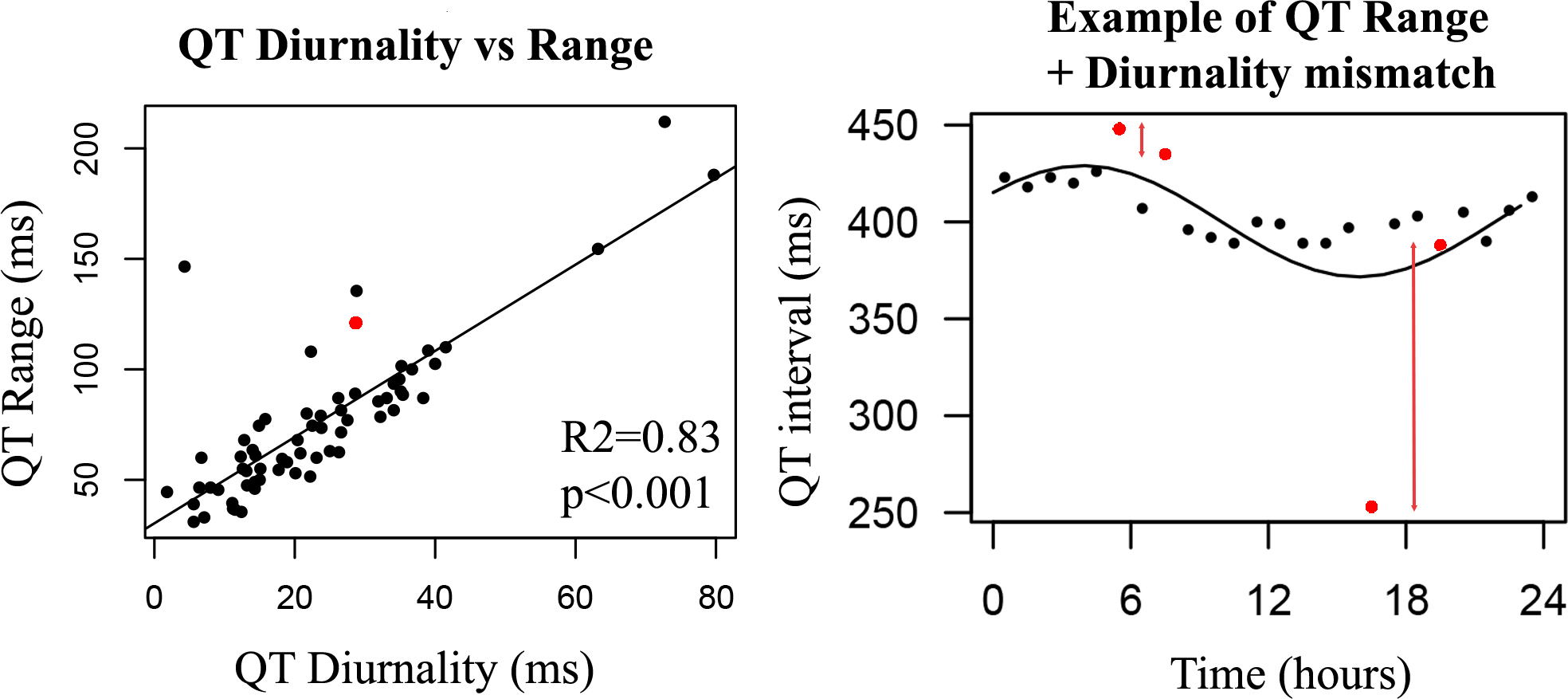

Supplement: Supplemental Figure 4 — Left: 24-h QT diurnality correlates with 24-h range. Red dot represents one of the outliers and is further analyzed in right side of figure. Right: 24-h rhythm in QT interval of example outlier. QT diurnality was calculated as difference between average and maximum of optimal cosine curve. QT range was calculated as difference between two maximal and minimal dots (depicted in red). The relatively high range in the example is caused by one very low value (red dot at bottom). [file Image4.JPEG]
